# Assessing the Role of Yarn Placement in Plated Knit Strain Sensors: A Detailed Study of Their Electromechanical Properties and Applicability in Bending Cycle Monitoring

**DOI:** 10.3390/s24051690

**Published:** 2024-03-06

**Authors:** Youn-Hee Kim, Juwon Jun, You-Kyung Oh, Hee-Ji Choi, Mi-Jung Lee, Kyeong-Sik Min, Sung-Hyon Kim, Hyunseung Lee, Ho-Seok Nam, Son Singh, Byoung-Joon Kim, Jaegab Lee

**Affiliations:** 1 Department of Convergence Design and Technology, Kookmin University, Seoul 02707, Republic of Korea; shell62@kookmin.ac.kr (Y.-H.K.); fbdk96@kookmin.ac.kr (Y.-K.O.); hichio1228@kookmin.ac.kr (H.-J.C.); 2 Department of Advanced Materials Engineering, Tech University, Siheung-si 15073, Republic of Korea; junjw98@naver.com; 3 School of Natural Sciences, Taejae University, Seoul 03151, Republic of Korea; mijung@taejae.ac.kr; 4 School of Electrical Engineering, Kookmin University, Seoul 02707, Republic of Korea; mks@kookmin.ac.kr; 5 Department of Fashion Design, Kookmin University, Seoul 02707, Republic of Korea; kim_sunghyon@kookmin.ac.kr; 6 Department of Fashion Industry, Incheon National University, Incheon 22012, Republic of Korea; srwalpha@inu.ac.kr; 7 School of Advanced Materials Engineering, Kookmin University, Seoul 02707, Republic of Korea; hsnam@kookmin.ac.kr (H.-S.N.); ssingh@kookmin.ac.kr (S.S.)

**Keywords:** conductive yarn placement, seamlessly integrated sensor, purl/knit plated sensors, contact resistances, wale-wise stretching, circuit modeling, bending cycle test

## Abstract

In this study, we explore how the strategic positioning of conductive yarns influences the performance of plated knit strain sensors fabricated using commercial knitting machines with both conductive and non-conductive yarns. Our study reveals that sensors with conductive yarns located at the rear, referred to as ‘purl plated sensors’, exhibit superior performance in comparison to those with conductive yarns at the front, or ‘knit plated sensors’. Specifically, purl plated sensors demonstrate a higher sensitivity, evidenced by a gauge factor ranging from 3 to 18, and a minimized strain delay, indicated by a 1% strain in their electromechanical response. To elucidate the mechanisms behind these observations, we developed an equivalent circuit model. This model examines the role of contact resistance within varying yarn configurations on the sensors’ sensitivity, highlighting the critical influence of contact resistance in conductive yarns subjected to wale-wise stretching on sensor responsiveness. Furthermore, our findings illustrate that the purl plated sensors benefit from the vertical movement of non-conductive yarns, which promotes enhanced contact between adjacent conductive yarns, thereby improving both the stability and sensitivity of the sensors. The practicality of these sensors is confirmed through bending cycle tests with an in situ monitoring system, showcasing the purl plated sensors’ exceptional reproducibility, with a standard deviation of 0.015 across 1000 cycles, and their superior sensitivity, making them ideal for wearable devices designed for real-time joint movement monitoring. This research highlights the critical importance of conductive yarn placement in sensor efficacy, providing valuable guidance for crafting advanced textile-based strain sensors.

## 1. Introduction

Textile sensors, which were integral for the development of smart garments, have attracted increasing attention for use in applications in human–device interfaces [1,2,3,4,5], human health and motion monitoring [6,7,8,9], sports analytics [10,11], soft robotics [12,13], and physical therapy [9,14]. These sensors can be seamlessly integrated into fabrics [6,15], thereby offering exceptional comfort and versatility. The boundaries of smart fashion are increasingly being pushed by the use of conventional textile techniques like weaving, knitting, braiding, and embroidery to create in-fabric sensors.

Knitted strain sensors are particularly useful in smart garment applications due to their inherent loop structure, which offers the flexibility and resilience needed for precise strain measurement. Moreover, the knitting process not only allows for design modifications in pattern and loop configuration to tune sensor sensitivity, but it also enables the easy integration of conductive yarns into fabrics, thus facilitating the development of scalable and robust smart garments [16].

Many studies have used plating techniques in knitting to create strain sensors. This approach, which is marked by its design flexibility [17,18,19] and robust processing capabilities [20], significantly enhances the sensors’ sensitivity, longevity, and resistance to washing [21,22]. A crucial consideration with these sensors is the interaction between the two types of yarns, which is largely influenced by the chosen placement of the conductive yarn within the knitting structure [20]. Specifically, the conductive yarn can be integrated into either the knit or purl stitches on the fabrics of the plated structures. Expertly combining these two fundamental stitches—knit and purl—in plated sensors makes it possible to engineer stitch patterns that are tailored to specific application needs.

Several notable studies have helped advance research in this area. For example, Atalay et al. [23] highlighted the vital link between base fabric attributes and sensor performance, while emphasizing the important effect that the selection of materials like elastomeric yarn and fabric design has on the functionality of knitted strain sensors. Xie et al.’s findings reveal that blending cotton with stainless steel yarn notably boosts the sensitivity, comfort, and durability of knitted fabric strain sensors, further highlighting the importance of material choice in wearable sensor design [24]. In another study, Raji et al. [25] examined the influence of different elastic yarn types and rib fabric structures on the efficacy of knitted underwear strain sensors. In two studies, Liu et al. [26] first provided insights into the linear relationship between the resistive properties of knitted fabrics and the proportion of conductive float and tuck stitches and then developed a geometric model integrated with a simplified resistive network; this model is key in determining the resistive impact of conductive float stitches in various knitted structures and calculating the equivalent electrical resistance in fabrics with floated stitches [27]. Warncke et al. [28] conducted a comprehensive investigation to develop drift-free elastic strain sensors with robust sensor signals for motion capture, considering various knit patterns and conductive yarn incorporations and the size of the strain sensors. This study is pivotal for understanding the cyclic electromechanical properties of weft-knitted strain sensors. Further, Liang et al. [29] introduced a size prediction model crucial for the fabrication of designed knit strain sensors, while Bozal et al. [30] shed light on the significance of conductive yarn positioning in rib-structured sensors developed using plating techniques.

Despite the extensive research investigating various factors influencing knit sensor properties—such as yarn types, stitch patterns, and dimensions—we still lack a comprehensive understanding of how these elements interact to affect sensor performance. Our study, therefore, aims to bridge this gap by focusing on the fundamental effects of basic stitches (knit and purl) and the interaction between conductive and non-conductive yarns. Elucidating these basics is expected to make a key contribution to the development of more complex stitch patterns in plated knit sensor designs [31,32].

In our research, we used a plated knit technique to create plain plated knit strain sensors, where the conductive yarn is positioned on the back (or front) side in the same manner as it is integrated into the purl (or knit) stitch on the fabrics. Our study focused on understanding how this specific yarn arrangement affected the sensors’ performance. We also use optical microscopy to observe how non-conductive yarns behaved under elongation, particularly in relation to the type of stitch used, i.e., knit or purl. These observations provided valuable insights into the relationship between stitch type and yarn interaction. Moreover, we developed a simple equivalent circuit model to estimate the impact of conductive interlocked and jammed loops on the contact resistance of these sensors. Our results indicated that positioning the conductive yarn at the back of the plated knit sensor (termed a ‘purl plated sensor’) led to markedly enhanced performance over the arrangement in which the conductive yarn was positioned at the front of the sensor (termed ‘knit plated sensor’), specifically in terms of higher gauge factor, improved stability, and responsiveness. We also demonstrate the practical applicability of these sensors in monitoring joint movements, supported by in situ data transmission and analysis. This research brings a novel perspective to smart textiles by delving into the fundamental aspects of stitch selection and yarn placement in plated knit strain sensors. The integration of circuit modeling, simulation, and cyclic bending tests marks a pioneering approach that could lead to enhanced sensor designs for tailored applications.

## 2. Experimental

### 2.1. Materials and Methods

For the strain sensor design, a plain knit design was chosen and knitted on a CMS330 KI W TT SPORT E7.2 (14 gauge) computerized flat knitting machine (STOLL, Reutingen, Germany) using a silver-coated nylon conductive yarn that was purchased from AMANN (Bonnigheim, Germany). This silver-coated nylon conductive yarns are chosen due to their superior electromechanical properties [33], unidirectional response to tension [25], and better suitability for wearability and durability [33] in practical strain sensor applications, compared with stainless steel-based fibers. Simultaneously, we chose a 50% wool and 50% acrylic blend for the non-conductive yarn, capitalizing on wool’s warmth, comfort, and insulating qualities, alongside acrylic’s flexibility, stretchability, affordability, and ease of maintenance. The silver-coated conductive yarn has an initial resistance of 530 Ω/m. Non-conductive yarn consisting of a 1:1 ratio of acryl and wool was also obtained from C&TEX (Seoul, Republic of Korea) and used as an insulating layer to make the knitted base fabric and plated sensors.

**Figure 1:**
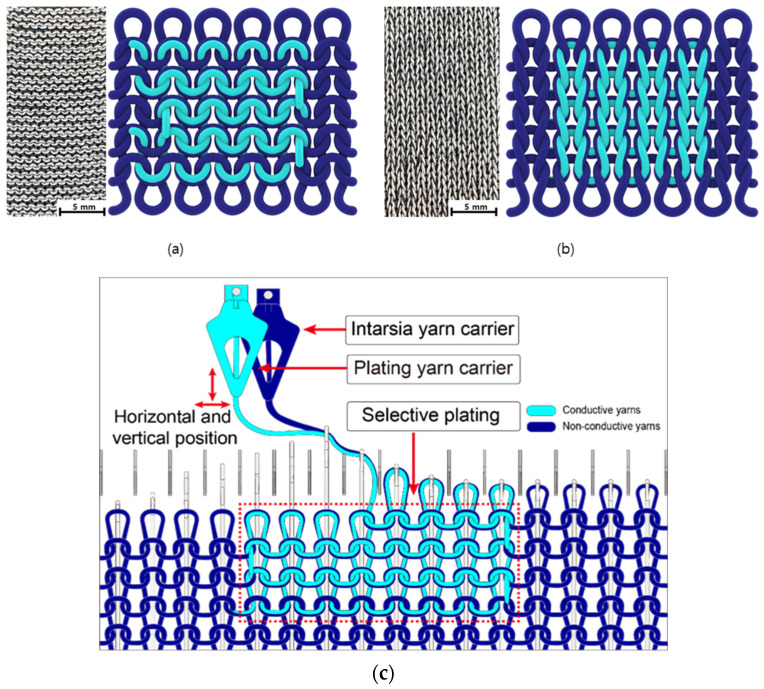
Illustration of machine-knitted textiles incorporating strain sensors, utilizing conductive yarns (light blue) and non-conductive yarns (dark blue). The figure demonstrates (**a**) the purl stitch pattern, (**b**) the knit stitch pattern, and (**c**) the plated knitting technique used for integrating the strain sensor. For parts (**a**,**b**), both the textile’s appearance and a schematic of the front side are shown.

Figure 1 illustrates two types of strain sensors integrated into machine-knitted fabrics, as shown in parts (a) and (b), using both conductive (light blue) and non-conductive (dark blue) yarns. The conductive yarn is managed by a plating yarn carrier and the non-conductive yarn by an intarsia yarn carrier, as detailed in part (c). The strategic modification of the carriers’ positions enables the creation of fundamental knit and purl stitches. For knit stitches, the conductive yarn is brought to the forefront, forming the visible ‘V’ pattern on the fabric’s surface as depicted in the schematic of (b). Conversely, the purl stitches bring the non-conductive yarn to the surface, creating the ‘bump’ texture shown in the schematic of (a). The selective positioning of yarns is dynamically orchestrated by alternating the position of the plating and intarsia carriers during the knitting process. This ensures the conductive yarn is knit into an integrated network of sensors within the fabric, which is primarily composed of non-conductive yarn.

The overall size of the sample including the non-conductive region and the conductive sensing area was 90 mm × 255 mm. The conductive yarn has a yarn count of Tex28, whereas the non-conductive yarn has a yarn count of Tex34 × 2. The conductive sensor’s course density was 14 courses/20 mm, and its wale density was 34 wales/40 mm. The size of the selectively plated sensors was 20 mm × 40 mm (14 courses × 34 wales).

### 2.2. Electromechanical Test Setup

#### 2.2.1. Measurement of Electromechanical Properties

A universal testing machine (UTM) with a 100 N load cell was used to generate a stretching of the knitted fabric sensors between 0 and 30% at a constant speed of 0.5 mm/s. A pair of clamps was used to fix the fabric in the wale direction. Textile samples for mechanical testing were prepared by cutting textiles to 40 mm (course) × 100 mm (wale) with a conductive sensing area of 20 mm (14 courses) × 40 mm (34 wales). The initial length of the fabric sample, which is known as the gauge length, was set at 80 mm. For the electromechanical tests, four alligator clips were attached to the ends of the conductive sensing area to measure the resistance using a four-point method where a constant current was applied to the two outer clips, and the resulting voltage drop was measured from the two inner clips. A digital multimeter system (KEITHLEY (DAQ6510, Keithley Instrument, Cleveland, OH, USA)) was used for real-time monitoring of the resistance during the stretching of the fabric sample.

#### 2.2.2. Durability Test: Cyclic Flexing

##### Test Equipment Description

**Figure 2:**
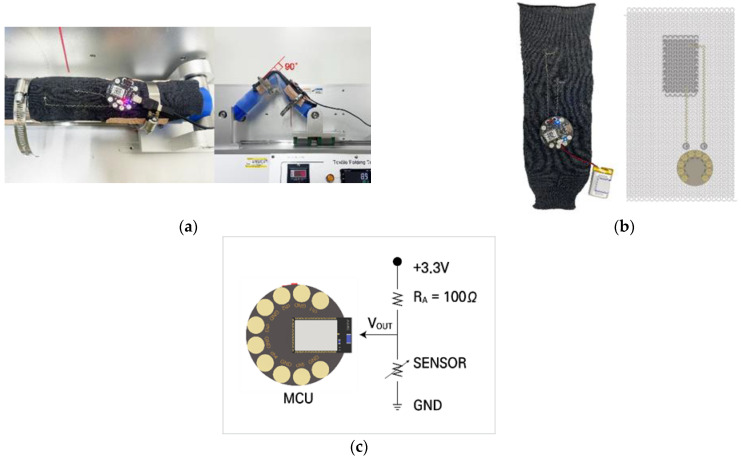
The experimental setup used to measure the dynamic bending resistance of the sample: (**a**) E-textile flexing tester; (**b**) wireless fabric sensor system consisting of a purl stitch plated sensor, an MCU module, and an interconnection module, and the inset (on the right side) shows a schematic of a fabric sensor system; and (**c**) a schematic of the voltage divider in the sensor system that shows the calculation of the voltage drop (Vout) across the sensor.

We evaluated the sample’s dynamic bending resistance using an E-textile flexing tester, model CKFT-T400 (Netest, Hwasung-si, Republic of Korea) as shown in Figure 2a. This device secured the sample on a cylindrical holder, ensuring it was firmly clamped at the ends for stability during testing. This holder, which simulates the movement of a human joint, has a diameter of 80 mm and is coated with a 3 mm thick silicone layer. The tested plated knit strain sensors, which were crafted on a knitting machine, measured 140 mm in the wale direction and 90 mm in the course direction. These sensors showed a specific sensing area of 20 mm by 40 mm, as illustrated in Figure 2b. The flexing tester could vary the bending angle from 0 to 135° and adjust the speed from 0 to 69 cycles per minute (cpm). In our tests, we consistently maintained a bending speed of 50 cpm and a bending angle of 90° across 1000 bending cycles.

##### Design of a Durable Wireless Electronic System for Testing

The fabric sensor system (Figure 2b), which is designed for potential commercial use, delivers accurate measurements and rapid response times. An accompanying schematic (shown in the inset of Figure 2b) demonstrates the front side layout of the fabric, which features a seamlessly integrated knit strain sensor, an interconnection module, and a microcontroller unit (MCU) module. The interconnection lines are constructed using highly conductive yarns with a resistance of 85 Ω/m. We used a lockstitch technique on the textile base to form two interconnection lines linking the sensor and the MCU, with the longer one having a resistance of roughly 10 Ω and the shorter one having a resistance of about 4 Ω.

One interconnection line extends from the sensor’s wale end to a male snap fastener that serves as the ground connection. The second line begins at the opposite wale end of the sensor, leading to another snap fastener that is supplied with a stable 3.3 volts. This configuration allows for the voltage variation to be monitored at two points on the sensor under dynamic conditions. We collected the sensor’s strain signals at 10 Hz via a voltage divider, which was governed by the following equation:

$$V_{OUT}=\frac{R_{2}}{R_{2}+R_{1}}\cdot V_{IN}$$

where *R*_1_ is the reference resistance of 100 Ω, *R*_2_ is the sensor’s variable resistance that changes with strain, *V_OUT_* is the sensor’s output voltage, and *V_IN_* is the input voltage of 3.3 volts.

### 2.3. Modeling of the Plated Knitted Strain Sensor

**Figure 3:**
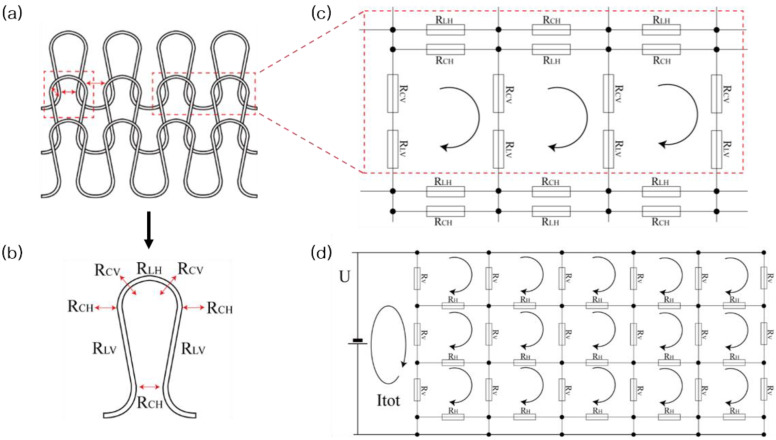
Circuit of unit loop and plated fabric network: (**a**) a schematic structure of the plated knit fabric; (**b**) a unit loop and its related resistances, (**c**) an equivalent resistance network circuit corresponding to the loop structure of 1 course × 2 wale, and (**d**) a simplified circuit.

Figure 3a–c illustrate the schematic structure of the plated knitted fabric, focusing on a unit loop within a dotted red box in Figure 3a’s left side, and a resistive network circuit for a 1 course × 2 wale unit structure, highlighted in a dotted red box in Figure 3a’s right side. This unit structure comprises two needle loops and one sinker loop. It features two types of contact resistance (*R_CV_*, *R_CH_*) and three length resistances (*R_LH_*, *R_LV_*), all outlined within a red dotted frame. *R_LH_* and *R_LV_* represent the length resistances of the needle loop and the limb, respectively, while *R_CV_* and *R_CH_* denote the contact resistances of the interlocked conductive loops and the jammed conductive loops, respectively [34]. The length resistance of a loop is influenced by its electrical resistivity and length, while the contact resistance is primarily influenced by the contact force [35].

A weft plain fabric consists of continuous loops that are interconnected in the course direction and intermeshed in the wale direction. The 1 × 2-unit loop, which is analogous to a fabric circuit network, as illustrated in Figure 3c, forms the basis for constructing a complete fabric circuit network. This network incorporates two types of resistors: vertical resistance (*R_V_*) and horizontal resistance (*R_H_*). *R_V_* is calculated as the sum of *R_LV_* and *R_CV_*, which are arranged in series along the wale direction. Meanwhile, *R_H_* is determined using the formula 1/*R_H_* = 1/*R_CH_* + 1/*R_LH_*, where *R_LH_* and *R_CH_* are connected in parallel in the course direction. Figure 3d illustrates a simplification of this circuit network.

The simplified circuit network calculates the equivalent resistance (*R*_eq_), which is defined as the ratio of applied voltage (*U*) to the total circuit current (*I*_tot_).

(1) $$R_{eq}=U/I_{tot}$$

Using Kirchhoff’s voltage law and MATLAB software (R2023b), the circuit network equations were established and solved to determine I_tot_ [35].

The relationship of the contact resistances (*R_CV_*, *R_CH_*) with the force applied to the fabric sensor and their relationships with *R_V_* and *R_H_* are crucial for simulating the equivalent resistance—the applied force curve. *R_CV_*, which is the contact resistance of vertically overlapped conductive yarns, is calculated using Holm theory [36], by which it is inversely proportional to the square root of the contacting normal force (F_N_):

(2) $$R_{CV}=B/\sqrt{F_{N}}$$

Here, B is assumed to be a constant. The contacting normal force (F_N_) depends on the loop configuration and dimensions, along with the applied force (F) on the fabric [35]. This relationship is expressed as follows:

(3) $$F_{N}=2\frac{F}{G\times N_{W}}$$

where N_W_ is the yarn number in the wale direction and G is a geometrical constant of the loops [35].

Consequently, R_CV_ can be defined as follows:

(4) $$R_{\mathrm{CV}}=C/\sqrt{F\;}$$

where C is a constant. Similarly, R_CH_, which is the resistance for horizontally jammed contacts, can be expressed using a similar formula with a different constant (C′):

(5) $$\;R_{\mathrm{CH}}=\;C^{\prime }/\sqrt{F}$$

Therefore, the equivalent vertical and horizontal resistances in the circuit model can be described as follows:

(6) $$R_{V}=R_{CV}+R_{LV}=C/\sqrt{F}+R_{LV}$$

(7) $$R_{H}=\frac{R_{CH}\times R_{LH}}{R_{CH}+R_{LH}}=\frac{(C^{\prime }/\sqrt{F})\times R_{LH}}{\left(C^{\prime }/\sqrt{F}+R_{LH}\right)}$$

These relationships were utilized in the simplified circuit network to calculate R_eq_ following the previously described process.

The calculations utilized several parameters, including geometrical factors such as the number of wales (*N_W_* = 100), wale number (14), and course number (27). Additionally, the geometrical constant *G* was determined based on measurements of loop geometrical dimensions: *L*_1_ (the length of a loop leg), *L*_2_ (the distance between the centers of overlapping loops), and *L*_3_ (course spacing) [35]. These measurements were essential for establishing the geometrical constant G. The influence of *R_V_* and *R_H_* on the applied force, alongside *R*_eq_, was assessed through circuit model simulations. Table 1 presents the input parameters used in these simulations, detailing the number of wales, courses, and specific loop geometrical factors, as well as the resultant output parameters for *R_V_* and *R_H_*, for both purl and knit sensors under varying applied force scenarios. It also includes the derived values of *R*_eq_. The comprehensive results of these simulations are elaborated upon in the Results Section.

## 3. Results and Discussion

### 3.1. Stretching Test

**Figure 4:**
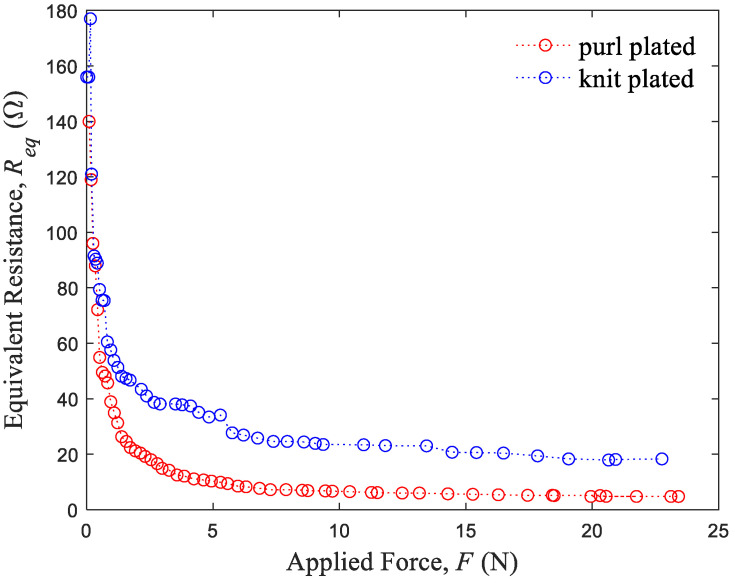
Resistance variations of the purl and knit plated sensors plotted as a function of applied force.

Figure 4 depicts the resistance behavior of purl and knit plated sensors under varying forces. Both sensor types demonstrate a marked decrease in resistance when subjected to forces up to approximately 1 N, after which they exhibit more gradual declines. Notably, the resistance of the purl sensor decreases more sharply in the initial stage of force application. This behavior suggests the existence of a more efficient contact between conductive yarns in the purl sensor, particularly under lower forces (below 1 N).

### 3.2. Simulated Results of Fabric Equivalent Resistance–Applied Force

**Figure 5:**
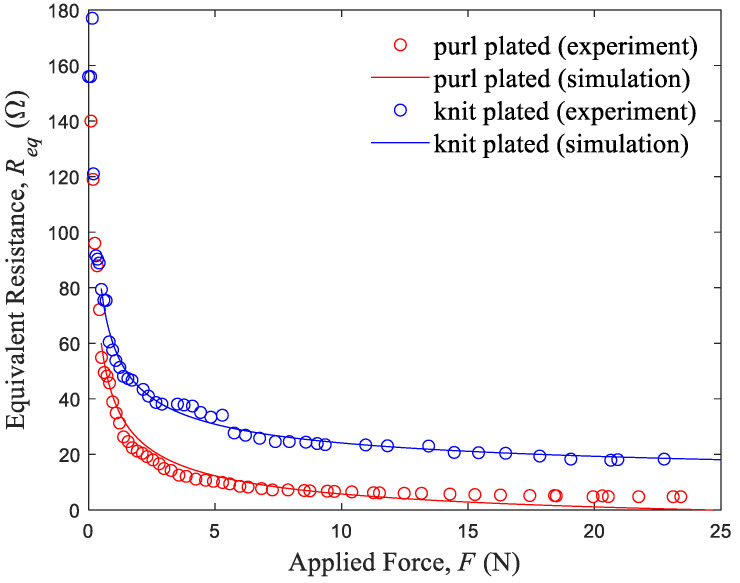
Simulated and experimental values of the plated sensors’ resistances plotted as a function of applied force.

**Figure 6:**
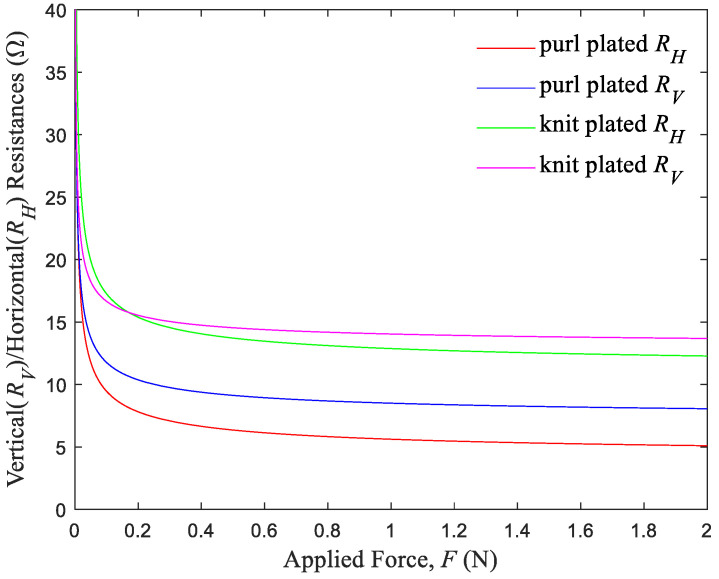
Variations in the vertical and horizontal resistances with forces applied to knit plated sensor and purl plated sensor, respectively.

The results of the simulation of fabric resistance, which is based on Kirchhoff’s voltage law and conducted using MATLAB software, correlate well with the experimental data (Figure 5). This correlation indicates that contact resistance significantly affects the performance of the plated knit strain sensors. Central to this simulation is a multiple linear regression model, which can be expressed as $R_{\mathrm{eq}}\approx 1+R_{V}R_{H}+R_{V}^{2}+R_{H}^{2}$. This model serves two purposes: first, it accurately fits the experimental curve of fabric resistance as a function of applied force (illustrated in Figure 6), thus quantifying the resistance changes under varying forces.

Secondly, the model elucidates the relationship between vertical and horizontal resistances (*R_H_* and *R_V_*) and the applied force (*F*), thus revealing how these directional resistances influence sensor performance.

Figure 6 details the specific relationships between vertical and horizontal resistances and the applied force for both sensor types. The resistance of the purl plated sensor is characterized by $R_{V}=1.50/\sqrt{F}+6.99$ and $R_{H}=1.77/\sqrt{F}+3.84$, while that for the knit plated sensor is characterized by $R_{V}=1.20/\sqrt{F}+12.82$ and $R_{H}=2.02/\sqrt{F}+10.84$. Notably, the purl structure exhibits significantly lower vertical and horizontal resistances compared to the knit structure, which contributes to the lower overall equivalent resistance of the purl sensor. Further, it is crucial to effectively control the contact resistances and the horizontal resistance to reduce the equivalent resistance of the plated knit strain sensors under strain in the wale direction. The predictive accuracy of these models is high, with R^2^ values of 97.9% for the purl sensor and 96.3% for the knit sensor, both of which explain over 96% of the variation in resistance. The models’ validity is further reinforced by the results of an F-test (F = 1477, *p* < 0.05), thus confirming their reliability in predicting changes in resistance under strain in the wale direction.

**Figure 7:**
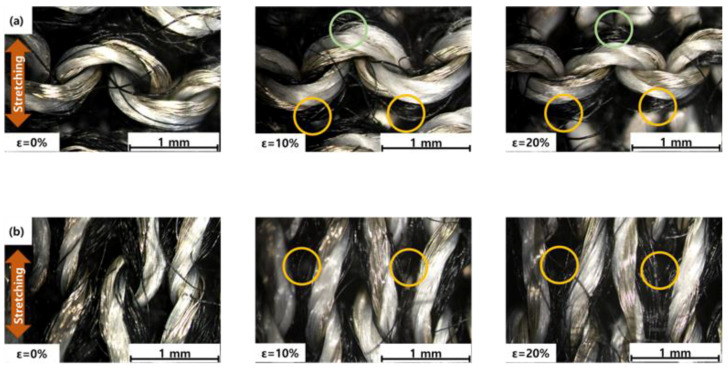
Optical microscopy images (**a**) of the purl plated strain sensor in the relaxed state, at a strain of 10% and at a strain of 20%, respectively; (**b**) corresponding images of the knit plated strain sensor in the relaxed state, at a strain of 10% and at a strain of 20%, respectively.

The behavior of non-conductive yarn in plated strain sensors was studied under varying loads using optical microscopy, with the results shown in Figure 7. Figure 7a demonstrates the purl plated sensor in both relaxed and elongated states. Key findings include the role played by non-conductive yarn among conductive yarns in determining the fabric’s mechanical properties. In a relaxed state, non-conductive yarns create noticeable separations and gaps in the fabric’s interlocked and jammed loops. Under a 10% strain, these yarns retract, thereby closing the gaps in a directional manner. Specifically, the non-conductive yarn located under a conductive needle loop rises, thus thickening the area above the conductive yarn (marked in green), while the yarn that is located under a conductive sinker loop descends (marked in yellow). This vertical movement is essential for enhancing contact between loops, as shown in the circled areas in Figure 7a. This pattern persists and becomes more pronounced at a 20% strain, indicating a consistent mechanical response.

By contrast, Figure 7b illustrates a different behavior in knit plated sensors, where non-conductive yarns move laterally instead of vertically. As the strain increases from 0% to 10%, these yarns are squeezed out from the conductive loops, eventually aligning parallel to them (marked in yellow). At 20% strain, the yarns continue to move laterally, thus thickening the areas between conductive yarns. This lateral displacement, which likely occurs in a non-uniform manner, impedes efficient contact between conductive loops, ultimately leading to less stable contacts compared to the vertical movement in purl plated sensors. This difference in yarn movement helps elucidate the structural and functional variations between purl and knit plated sensors under strain.

**Figure 8:**
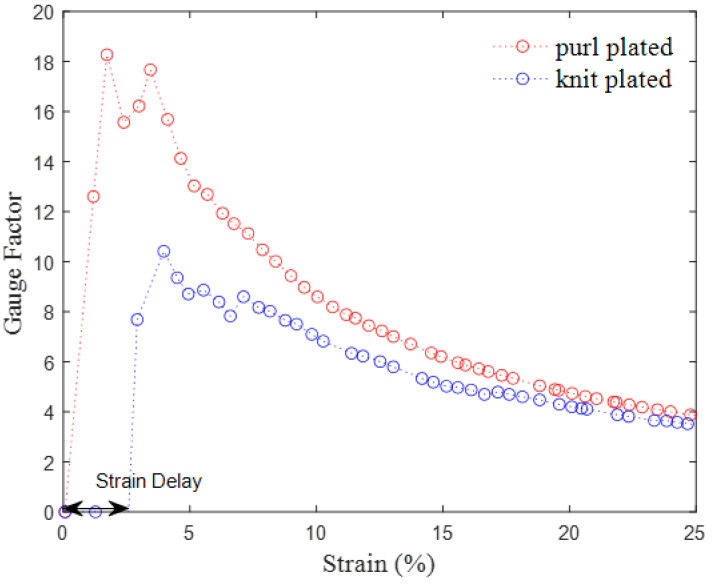
The variation in the gauge factor of the purl and knit plated sensors as a function of strain.

The sensors’ sensitivity, which is quantified in the form of the gauge factor (GF), is explored in Figure 8. The GF is defined as the ratio of fractional change (∆*R/R*) in electrical resistance to the strain (ε) in the stretching direction of the sensor [33].

(8) $$\mathrm{GF}=\frac{\Delta R/R}{\varepsilon }$$

With gauge factors ranging from 3 to 18, the purl sensor demonstrates a shorter strain delay (1% strain) than the knit sensor (3% strain), which might indicate a faster response at low strain levels.

### 3.3. Dynamic Bending Test of Textile Sensors

Given the complex and dynamic strains induced by human movements, durability is a critical factor for wearable strain sensors. In particular, it is essential for these sensors to maintain their electromechanical properties without degradation or failure caused by fatigue deformation of the active materials.

For this reason, a durability test was conducted using a wireless strain sensing system (illustrated in Figure 2) designed for bending applications, such as real-time monitoring of joint movements (e.g., fingers, elbow, body joints).

**Figure 9:**
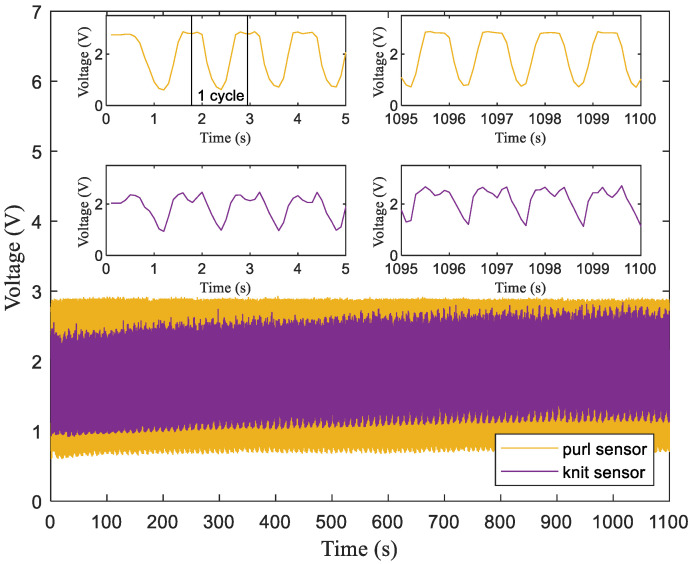
Output voltage changes of a 20 mm × 40 mm fabric sensor during the repetitive bending processes at 50 cpm.

Figure 9 presents the output voltage variation over 1000 bending cycles for two different sensor structures. The insets show the strain–output voltage curves for four bending–recovery cycles at the initial and final stage of bending, thus highlighting the elapsed time–output voltage curves for single bending–recovery cycles of the purl plated fabric sensor and the knit plated fabric sensor. The insets also show the behaviors of one bending cycle of the sensors. During bending, the output voltage initially increases, after which it rapidly decreases as the fabric extends further. At maximum fabric elongation, the voltage drops to its lowest point. During recovery, as the fabric relaxes, the voltage first increases and then decreases [8]. This variance in the output voltage at different stages of bending and recovery is attributed to the strain delay in the fabric, thus reflecting the hysteresis of the devices.

Notably, compared to the knit plated variant, the purl plated sensor demonstrates superior performance with less delay, higher sensitivity, and most importantly, greater reproducibility. As demonstrated in Figure 9, the output voltage remains remarkably consistent after 1000 bending cycles, underscoring the sensor’s exceptional reproducibility, quantified by a low standard deviation of 0.015 [37]. This contrasts markedly with the knit strain sensor’s higher standard deviation of 0.118, highlighting the purl plated sensor’s superior stability and reliability in performance measurements. In addition, the amplitude of this curve correlates with the device’s gauge factor. For example, the purl plated fabric demonstrates a voltage gauge factor, which is calculated as the ratio of the normalized voltage change to the strain. This factor is 3.15, as determined by (2.87 − 0.61)/(2.87 × 0.25), whereas the knit plated fabric has a factor of 2.46, as determined by ((2.44 − 0.94)/(2.44 × 0.25)). These calculations were made under conditions where a 25% strain was measured during 90° bending.

The dynamic response characteristics of the purl plated sensor were also assessed by evaluating its response time. During the bending and relaxing states, the purl strain sensor exhibited response times of approximately 350 ms and 250 ms, respectively. Meanwhile, the knit strain sensor displayed response times of around 250 ms for bending and 300 ms for relaxing. Given the typical use of textile strain sensors in wearable electronics, which is to monitor human body motion and various external stimuli, these response times are considered to be sufficient for real-time detection [19,38].

Operational stability was assessed through 1000 cycles of 90° bending, and the results revealed that the purl sensor operated stably without degradation, as depicted in Figure 9, thus indicating that the purl plated knit strain sensor possesses commendable operational stability for practical applications.

## 4. Conclusions

In the present research, we examined how different stitching types—specifically purl and knit—affect the performance of plated knit strain sensors. To this end, we developed a simple equivalent circuit model that accounts for both horizontal and vertical resistances. This model helped us understand the impact of contact resistances, both from jamming and interlocking contacts, on the overall resistance of the fabric sensors. Our MATLAB simulations highlighted the significant role played by horizontal contacts in decreasing resistance during loading and unloading in knit fabrics. Interestingly, we found that the purl stitch results in a lower contact resistance compared to the knit stitch. This might be attributable to the unique vertical movement of non-conducting yarn in the purl structure, which facilitates easier contact between adjacent conductive loops than the lateral movement observed in the knit structure.

To explore the practical applicability of such an arrangement, we designed a plated knit sensor system capable of in situ monitoring of bending motions. This system, which was equipped with machinery enabling real-time observation of bending cycle tests, demonstrated notable improvements in the purl structure’s performance. Compared to the knit structure, the system using the purl structure’s attributes, such as sensitivity, stability, and strain delay, were found to be significantly enhanced with a reasonable response time. Altogether, these findings suggest that the purl stitch-based sensor could serve as an effective platform for commercial wearable strain sensors.

## Figures and Tables

**Table 1:** Parameters and results from simulation models.

Input Parameters	Output Parameters
*N_W_* = 100	*R_V_* $:\;\mathrm{Purl}\;\mathrm{Sensor}:\;1.50/\sqrt{F}+6.99\;$
Wale Number: 14	$\mathrm{Knit}\;\mathrm{Sensor}:\;1.20/\sqrt{F}+12.82$
Course Number: 27	*R_H_* $:\;\mathrm{Purl}\;\mathrm{Sensor}:\;1.77/\sqrt{F}+3.84\;$
Loop geometrical factors:	$\mathrm{Knit}\;\mathrm{Sensor}:\;2.02/\sqrt{F}+10.84$
*L*_1_ = 1.5 mm, *L*_2_ = 1.0 mm, *L*_3_ = 2.0 mm	*R*_eq_

## Data Availability

Data are contained within the article.
